# Phased Feature Extraction Network for Vehicle Search Tasks Based on Cross-Camera for Vehicle–Road Collaborative Perception

**DOI:** 10.3390/s23208630

**Published:** 2023-10-22

**Authors:** Hai Wang, Yaqing Niu, Long Chen, Yicheng Li, Tong Luo

**Affiliations:** 1 School of Automotive and Traffic Engineering, Jiangsu University, Zhenjiang 212013, China; 2 Automotive Engineering Research Institute, Jiangsu University, Zhenjiang 212013, China; 3 School of Automobile and Traffic Engineering, Jiangsu University of Technology, Changzhou 213001, China

**Keywords:** VICAD, vehicle search, datasets, automatic driving, over-the-horizon perception

## Abstract

The objective of vehicle search is to locate and identify vehicles in uncropped, real-world images, which is the combination of two tasks: vehicle detection and re-identification (Re-ID). As an emerging research topic, vehicle search plays a significant role in the perception of cooperative autonomous vehicles and road driving in the distant future and has become a trend in the future development of intelligent driving. However, there is no suitable dataset for this study. The Tsinghua University DAIR-V2X dataset is utilized to create the first cross-camera vehicle search dataset, DAIR-V2XSearch, which combines the cameras at both ends of the vehicle and the road in real-world scenes. The primary purpose of the current search network is to address the pedestrian issue. Due to varying task scenarios, it is necessary to re-establish the network in order to resolve the problem of vast differences in different perspectives caused by vehicle searches. A phased feature extraction network (PFE-Net) is proposed as a solution to the cross-camera vehicle search problem. Initially, the anchor-free YOLOX framework is selected as the backbone network, which not only improves the network’s performance but also eliminates the fuzzy situation in which multiple anchor boxes correspond to a single vehicle ID in the Re-ID branch. Second, for the vehicle Re-ID branch, a camera grouping module is proposed to effectively address issues such as sudden changes in perspective and disparities in shooting under different cameras. Finally, a cross-level feature fusion module is designed to enhance the model’s ability to extract subtle vehicle features and the Re-ID’s precision. Experiments demonstrate that our proposed PFE-Net achieves the highest precision in the DAIR-V2XSearch dataset.

## 1. Introduction

A vehicle search involves locating and identifying vehicles in uncropped images of the real world. It has a wide range of applications in intelligent transportation systems and has become essential to the realization of autonomous driving as a result of the continuous development of technology. For the vehicle search task, a comprehensive, trustworthy, and objective dataset is conducive to objectively evaluating the performance of an algorithm, which is one of the most important aspects of the entire task. However, there is no suitable vehicle search dataset.

Observing the existing pedestrian search [1] datasets, roadside cameras frequently use these datasets as data acquisition methods to address security concerns. In accordance with the adage “stand high, see far”, the data captured by the roadside camera is typically less obscured and shot more steadily. Nevertheless, in autonomous driving scenarios, roadside cameras frequently have two deficiencies: (1) The roadside cameras can only capture a single angle of the vehicle target. (2) The roadside camera cannot track the long-term target, and it is challenging to fully extract the features of the foreground target.

Moreover, vehicle cameras are primarily used for data acquisition in modern automatic driving [2]. However, the camera mounted on the side of the vehicle frequently encounters issues, such as occlusion, that prevent it from achieving environmental perception without dead corners. Therefore, Bishop et al. [3] argue that single-vehicle intelligence is not an effective solution to the autonomous driving problem. Consequently, numerous vehicle–road collaboration technologies [4,5] have emerged. Collaboration between vehicles and roads refers to the cooperation between vehicles and roads. The infrastructure is used to provide vehicles with information that extends well beyond their current field of view so that they can complete tasks such as target detection and trajectory prediction, which will ensure future control decisions are correct and safe. If vehicle–road collaboration technology is added to the vehicle search task in order to increase the capability of comprehensive perception, the acquisition ability of vehicle targets and the training precision of the model will be improved.

Based on previous research, the DAIR-V2XSearch vehicle search dataset has been developed. Based on the vehicle–road collaboration DAIR-V2X dataset [6] proposed by Tsinghua University, this dataset is compiled. The vehicle is selected and matched, then the vehicle ID and camera ID are labeled using the data collected by both ends of DAIR-V2X and the tagged vehicle anchor. The DAIR-V2XSearch dataset is advantageous in the following ways: (1) By utilizing the vehicle camera as the mobile end and the roadside camera as the fixed end, the roadside camera can be compensated by the vehicle camera, resulting in a more comprehensive perspective of the same vehicle. (2) Diverse backgrounds are collaborated to produce by two devices. Additionally, the two devices are installed at different heights, so the same vehicle captured from the same vantage point may appear slightly different. (3) Unlike large-scale datasets with good annotations generated in virtual scenarios (such as Sim4cv [7], Carla [8], and other simulators), this dataset is obtained in real-world scenarios, compensates for actual errors caused by virtual scenarios, and facilitates subsequent groundwork.

**Figure 1:**
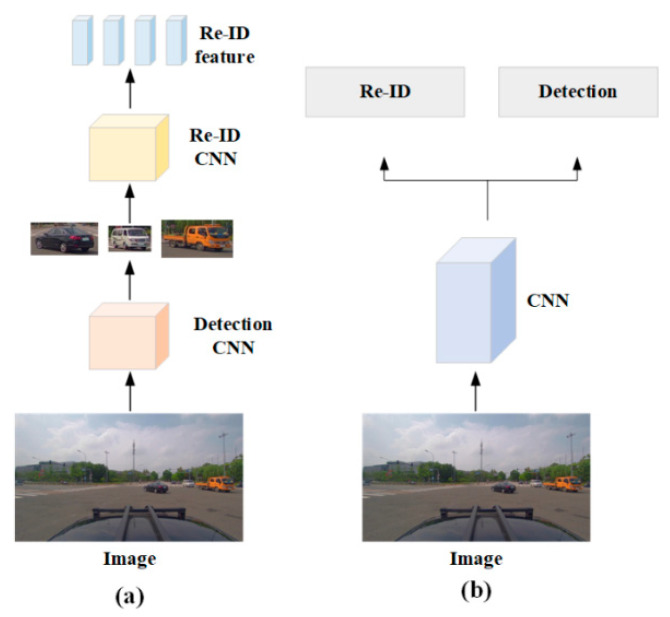
Search task framework diagram: (**a**) two-step model structure and (**b**) one-step model structure.

Existing vehicle search algorithms [9,10,11,12,13] continue to face extremely challenging retrieval and fine-grained problems. In addition to accurately locating the vehicle in the image, the vehicle in the background should also be distinguished and identified in the vehicle search. Currently, there are two categories of technology: one-step and two-step. As shown in Figure 1a, the two-step formula [9] is divided into detection and Re-ID, two independent tasks. First, the existing detection model is used to locate the vehicle, followed by the transmission of the cropped vehicle box to the Re-ID network in order to extract the subtle differences between vehicles. The two-step method can achieve high levels of precision, but it is time-consuming and computationally intensive. As a result, the one-step [10,11,12,13] method was developed. This approach combines detection and Re-ID from beginning to end, as depicted in Figure 1b. The Faster R-CNN framework is utilized in the majority of current one-step models for detection [14], with Re-ID branches added to complete the search task.

The reasons why the accuracy of the one-step method cannot be improved are as follows. First, the anchor boxes are responsible. Anchor boxes are initially designed for target detection [14] and have been employed in Faster R-CNN. However, anchor boxes cannot be utilized to extract Re-ID features. Many fuzzy features are introduced into Re-ID training because anchor box training frequently involves one box corresponding to multiple vehicle IDs or multiple anchor boxes corresponding to one vehicle ID. The second cause is the shared functionality between the two tasks. The detection task is the classification of a class, while the Re-ID task is the classification of multiple ids that belong to the same class. If two tasks utilize identical features, each task’s performance may suffer.

Therefore, a new network, the phased feature extraction network (PFE-Net), is proposed that effectively addresses the aforementioned problems. This network is based on the YOLOX [15] one-stage detection network, which is designed without anchor frames and has a high detection rate. Unlike previous “detection first” [16,17] or “Re-ID first” [18] frameworks, detection and Re-ID tasks have been equally treated in our architecture. Re-ID includes two isomorphic branches for detection and feature extraction. The detection branch is implemented as an anchor-free box, and SimOTA advanced label assignment is the candidate label assignment strategy. By performing the Re-ID operation on each pixel, the pixel-centered object is represented by the Re-ID branch. In order to better adapt to the produced vehicle search dataset, a camera grouping module and a cross-level feature extraction module are also proposed.

The three most significant contributions of this paper are as follows:

1. To address the insufficiency of vehicle search datasets, a collaborative vehicle search dataset for real-world vehicle scenarios, DAIR-V2Xsearch, is developed.
2. To complete the vehicle search more efficiently, a network for phased feature extraction is designed. Combined with the characteristics of the vehicle itself, two modules are simultaneously designed.
3. In order to validate the performance of the model, it is included in the DAIR-V2Xsearch dataset for a large number of experiments, and its performance reaches the highest level. Simultaneously, experiments are conducted on the pedestrian search dataset PRW to validate the generalization of the model, achieving high accuracy.

## 2. Related Studies

### 2.1. Vehicle Search

The objective of vehicle search is to complete the task of locating and identifying the same vehicle given a vehicle target from an uncropped, real set of images, which is the union of the two tasks of vehicle detection and Re-ID. In recent years, pedestrian search has developed rapidly and achieved remarkable results [11,12,13]. Consequently, vehicle searches are also slowly evolving. As there are few studies on vehicle search, pedestrian search is the primary research focus. The current framework for pedestrian search can be divided into two-step and one-step modes. A two-step procedure was employed by Zheng et al. [1]: first, vehicles were detected, then the obtained detection box was inserted into the Re-ID network, and finally, the result was obtained. Although the precision of the final search is high, the model was too large and complex, and the calculation speed was slow. The online strength matching loss was created by Xiao et al. [12] for Re-ID calculations, and the first one-step mode based on Faster-RCNN was proposed. A new Re-ID cut layer was added after the detection features to perform Re-ID matching and calculate the loss. In this way, not only speed calculations but also accuracy were improved. Norm-aware embedding was proposed by Chen et al. [11] to embed pedestrians within detection norms and Re-ID angles, respectively. Despite this improvement, the search frame continued to utilize the original two-stage anchor-base detection network, and the speed remained slow. Subsequently, the first one-stage anchor-free model proposed by Yan et al. [18], with an alignment feature aggregation module designed to adhere to the Re-ID first principle, was found to improve efficiency without sacrificing accuracy. Inspired by previous research, a new similar one-stage anchor-free framework is designed for the vehicle search model, which simultaneously trains the detection and Re-ID tasks. In addition, two new modules are designed based on the characteristics of the vehicle to improve the suitability of the model for vehicle feature extraction.

### 2.2. Vehicle Search Dataset

In recent years, numerous pedestrian search datasets have been published. PRW [1] consisted of data collected by six roadside cameras, while the bounding box position and pedestrian ID were manually labeled. VeRi776 [19] was a vehicle Re-ID dataset obtained by photographing a one-square-kilometer area in 24 h while restricting vehicles to predefined bounding boxes. Recent research on autonomous driving reveals that single-vehicle perception was plagued by occlusions, but these shortcomings could be compensated by the cooperative perception of vehicle and road. DAIR-V2X [6] was the first real-world vehicle–road collaboration dataset annotated with category information and bounding boxes. In general, there is no dataset dedicated to the vehicle search task. Hence, a cross-camera vehicle search dataset, DAIR-V2XSearch, is created to complete the task more effectively.

### 2.3. Vehicle Re-ID

**Figure 2:**
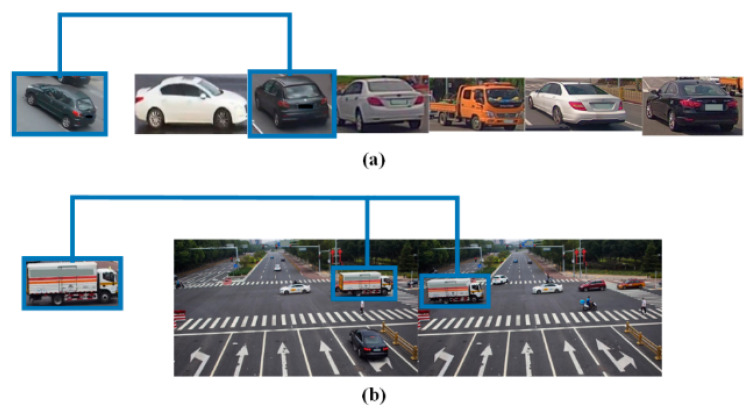
Comparison of the vehicle Re-ID and vehicle search dataset. (**a**) is a Vehicle Re-ID dataset, and (**b**) is a Vehicle Search dataset.

Vehicle Re-ID refers to the process of learning embeddedness features from cropped vehicle images, which is a significant distinction from vehicle search tasks. As shown in Figure 2, (a) is the form of the vehicle Re-ID dataset, and (b) is the form of the vehicle search dataset. In recent years, vehicle Re-ID has been extensively studied. Some methods [20,21] were primarily used to extract easily identifiable vehicle features with a high degree of precision. However, individual, easily identifiable feature information must be marked during training, which requires a significant amount of manpower. There are also methods for feature extraction that achieve high accuracy by designing measurement models [22], adding attention mechanisms [23], generating adversarial networks [24], etc. The conflict between detection and Re-ID tasks is analyzed in vehicle search, two tasks in parallel are processed, and a hierarchical feature extraction module is designed to improve the training accuracy in the Re-ID branch.

### 2.4. Vehicle Detection

There are two classification methods for existing vehicle detection techniques. Firstly, based on the process, they can be divided into: (1) Two-stage methods: This approach typically involves an intermediate region, such as Faster RCNN [14], MASK RCNN [25], etc. As it requires calculating candidate regions, it consumes a lot of memory and reduces detection speed. (2) One-stage methods: This approach outputs the detection results, including YOLO [26], SSD [27], etc., without generating region proposal boxes. This method is fast, and, with continuous improvement, its accuracy can compete with that of two-stage methods.

Secondly, based on the design method of the anchor box, they can be divided into the following. (1) Anchor-based methods: To obtain the best detection performance, typically, clustering analysis needs to be performed on the anchor points in the dataset before training to determine a set of optimal anchor points. This is a complex process that introduces some prior knowledge to the network. Existing datasets typically require a lot of experiments to determine the optimal anchor points. (2) Anchor-free methods: Anchor-free detection methods [15,28] do not require anchor boxes and have a simple structure and fast calculation speed, such as CornerNet, YOLOX [15], etc. As this is a new dataset, clustering and analysis need to be performed on the dataset to obtain previous boxes, which makes the process complex. The existing anchor-free single-stage detection network does not require this process. To simplify task completion and make the designed model more suitable for different datasets, the anchor-free detection network YOLOX [15] is selected as the basic framework for vehicle search.

## 3. DAIR-V2XSearch Dataset

### 3.1. Data Acquisition

In autonomous driving, vehicle cameras are used for a variety of purposes. However, numerous studies have found that single perception is frequently hampered by occlusion and other issues. Under vehicle–road cooperation, a vehicle search dataset is created to enhance the performance of vehicle searches. This vehicle search dataset is a modification of the DAIR-V2X dataset proposed by Tsinghua University. This section provides a comprehensive overview of the dataset for cooperative over-the-horizon perception of vehicle and road task requirements.

**Figure 3:**
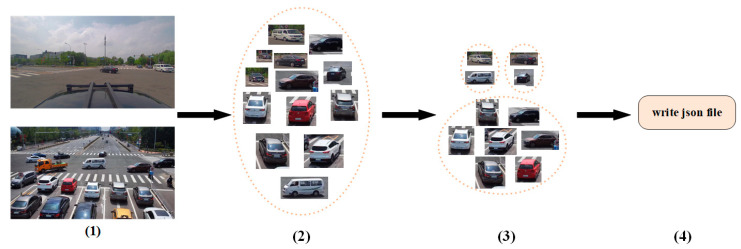
Dataset production process. (**1**) Match the time of the two camera ends, (**2**) Obtain the vehicle boundary boxes, (**3**) Match the same vehicle ID, (**4**) Write json file.

(1) Sensors: The dataset is collected at 28 intersections selected from Beijing’s autonomous driving demonstration zone, with four pairs of high-resolution cameras deployed as roadside devices at each intersection to collect data from various perspectives. In addition, a front-view camera is installed on the vehicle as a vehicle-end device in order to complete the acquisition simultaneously.

(2) Data processing: Due to the fact that the two devices jointly perform vehicle searches, it is necessary to time-match the data collected by the two devices. If the time difference between the two devices’ data is less than 10 ms, the collected data are selected, and the synchronization time is recorded. The captured video data was then used to crop the keyframes with a 10 ms time difference.

(3) Data labeling: An ID is identified and assigned to the vehicle in the cropped image. In addition to including the camera ID annotation, the vehicle camera ID is set to 0, and the roadside camera ID is set to 1. Each vehicle identification number is associated with at least one camera device. In total, 492 vehicle identification numbers are annotated, each of which is annotated at least twice. As shown in Figure 3, this is the production process and visualization of the dataset. First, the time between two devices is matched, and the vehicle target is clipped. Then, the same vehicle and ID assignment are identified. Finally, information, including the bounding box and vehicle ID, is written in a JSON file. Following the existing sample distribution convention for pedestrian search datasets, the images are divided into two sub-datasets, train and gallery, with a ratio of 1:2, and a trimmed box is randomly selected from each vehicle ID contained in the gallery to form the query dataset. The train dataset is used for training, while the gallery and query datasets are used for testing.

### 3.2. Dataset Contributions

(1) The initial search vehicle dataset: Using the dataset in two contexts, research is conducted to improve the applicability of vehicle search technology to the field of autonomous driving. Not only the issue of data occlusion caused by vehicle camera acquisition, but also the issue of limited shooting range caused by the roadside camera’s fixed field of view are effectively addressed by this method.

(2) Provide complex environmental information: Complex environmental information is contained in the vehicle search dataset. Data is collected by two devices from different angles, which results in data with variable backgrounds, resolutions, and perspectives. The model’s robustness has improved and is more suitable for tasks such as vehicle cross-camera object tracking [29], trajectory prediction [30], and others.

The dataset’s annotation and additional specific information have been added to the website. Download the dataset at https://github.com/Niuyaqing/DAIR-V2XSearch.git (accessed on 26 February 2023).

## 4. Methodology

### 4.1. Review

**Figure 4:**
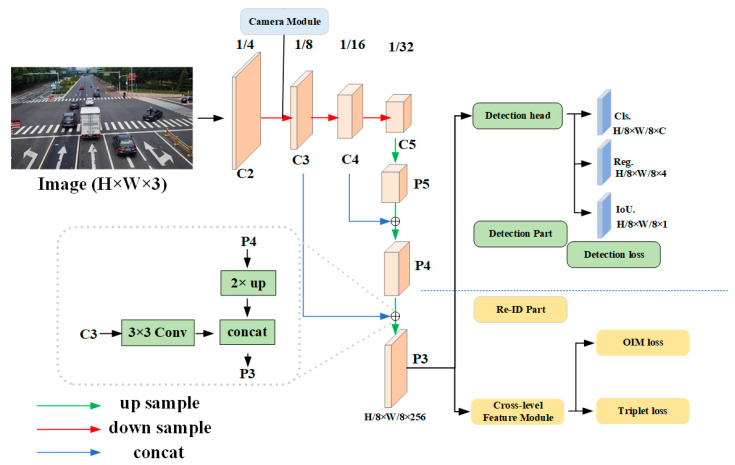
Phased feature extraction network structure diagram uses two parallel branches for the two sub-tasks of detection and Re-ID. The specific structures of the two branches are shown subsequently. The camera grouping module is received in the backbone.

To meet the requirements of vehicle search under vehicle–road collaboration, the phased feature extraction network for vehicle search is introduced in this section. The network structure is shown in Figure 4. In Section 4.2, the benefits and drawbacks of anchor-free and anchor-base networks are analyzed, and YOLOX is chosen as the best backbone network for vehicle search. Then, in Section 4.3 the network for detecting branch parts is designed, and the detection head is decoupled in order to improve the vehicle’s detection accuracy. Finally, in Section 4.4, the Re-ID branch is introduced, which uses the design of the camera grouping module and the feature stratification module to extract small, fine-grained differences between vehicles in order to improve the precision of the vehicle search.

### 4.2. YOLOX Network

YOLOX is one of the most popular one-stage anchor-free object detection methods because both large and small objects without anchors are detected. For a moving vehicle, the boxes from a distance are obtained by using the roadside camera, which will drastically alter the box and increase the applicability of the anchor-free detection method. In addition, for the anchor-base, a single anchor box may correspond to multiple IDs, or multiple anchor boxes may correspond to a single ID, introducing a great deal of ambiguity during the training of Re-ID features, which is not optimal for training the model. In addition, excellent detection accuracy is provided by YOLOX. Despite the fact that object detection focuses on acquiring inter-class information and Re-ID focuses on differentiating inter-class information, there is a conflict between the two tasks that makes learning them simultaneously challenging. However, a more precise box for the detected sample produces a higher detection accuracy, which can result in a higher Re-ID accuracy.

Afterward, two tasks, vehicle detection and vehicle Re-ID, are simultaneously preformed. The YOLOX detection head is used for detection, and excellent accuracy is achieved. The two designed modules are then added to the vehicle search task to complete it. The specific network model framework is shown in Figure 4.

### 4.3. Detection Branch

In object detection, classification and regression tasks frequently conflict with each other, which is a well-known issue [31]. In this section, the YOLOX detection head is employed. The detection head is set to a decoupled structure, and the regression and classification are output separately, which significantly accelerates the model’s convergence.

(1) Creating Corresponding Alignment Entities

**Figure 5:**
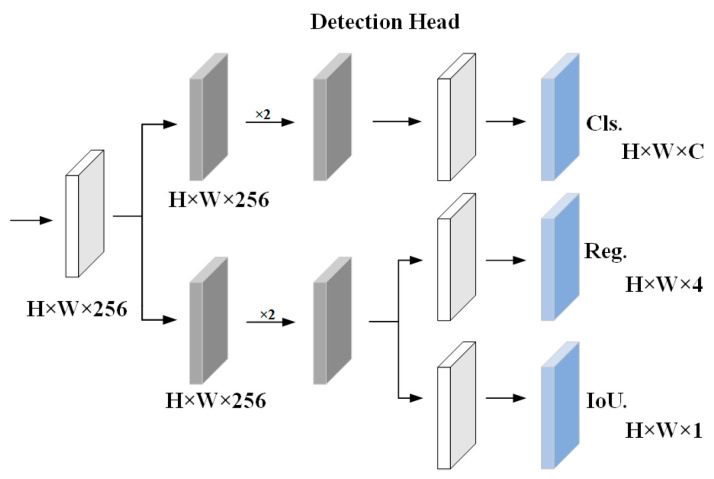
Structure diagram of the detection branch.

In the original YOLOX model, different levels of features are used to detect objects of different sizes, which significantly improves the detection accuracy. However, for the Re-ID task, because the Re-ID features obtained at different stages are distinct, there are different background features, which have a significant impact on the learned discrimination ability. The complexity of the model and the slowdown in training are also increased by using multiple stages for detection, neither of which is conducive to the subsequent Re-ID task. Even though the low-order feature has less semantic information, sufficient location information is contained. Therefore, the detection framework based on FPN [32] is modified, low-order and high-order features are combined, and detection with a single detection head is performed. The structure of the detection head is shown in Figure 5.

To connect the two parts laterally, the $\left\{C_{3},C_{4},C_{5}\right\}$ feature network from the Resnet-50 backbone is utilized, and then each stage is upsampled to obtain the $\left\{P_{3},P_{4},P_{5}\right\}$ feature network. Here, a 3 × 3 deformable convolution is employed, which can better adapt and adjust the receptive field on the input feature map to produce increasingly precise feature maps.

(1) $$P_{3}=∁\left(conv\left(P_{4}\right),conv\left(C_{3}\right)\right)$$

where two 1 × 1 convolutions are used at $P_{4}$ and 3 × 3 convolutions are used at $C_{3}$. ∁ is represented as the concatenation of two features for improved multi-level feature aggregation. In order to achieve a good balance between the performance of the two subtasks of detection and Re-ID, the largest feature generated at $\left\{P_{3}\right\}$ is only used for detection, ignoring a certain detection performance. The specific results are detailed in Section 4.3.

(2) Detection Loss Calculation

*GIoU_loss_* is used to calculate the confidence *IoU_loss_* when calculating the detection branch’s loss.

(2) $$L_{GIoU}=IoU-\frac{\left|C\backslash (A\cup B)\right|}{\left|C\right|}$$

where A and B are boxes for calculating IOU, and C is the outermost box of A and B.

*BCE_loss_* (Binary CrossEntropy loss) is utilized by the detection box position loss, *Obj_loss_*, and the classification loss, *Cls_loss_*.

(3) $$L_{BCE}=-y\times log\left(\tilde{r}\right)-\left(1-y\right)\log\left(1-\tilde{r}\right)$$

where r is represented as the model output value, whose size must be between 0 and 1, and y is represented as the real label.

### 4.4. Re-ID Branch

As part of class-based feature comparison, the Re-ID branch is used to extract more discriminative features between vehicles. To accomplish this objective, two modules are designed that address the Re-ID branch separately.

(1) Camera Grouping Module

Typically, the dataset for a search task is collected from multiple cameras. Due to the use of multiple cameras, multiple perspectives of the same vehicle can be obtained. However, due to the varying installation positions of the cameras, the pictures they capture will result in significant differences in color, saturation, and brightness. As a result, a camera embedding module is proposed that employs camera ID for simple grouping and imparts camera information into features for aggregation in order to distinguish internal differences between cameras. The insertion position of the camera grouping module is shown in Figure 4.

Specifically, the dataset contains N cameras, denoted as ${ID}_{r},\;r\in [1,N]$. To initialize the module, a randomly generated sequence is utilized. Following initialization, the camera embedding is obtained as $E_{c}\in R^{N_{C}\times A}$, where A=H×W, and *H* and *W* are represented as the height and width of the corresponding image in the current $V_{0}$ channel, respectively. The corresponding camera embedding feature for a photo $img_{i}$ captured by a camera $ID_{r}$ can therefore be expressed as $Ec_{i}^{r}$. The camera embedding feature $E_{c}$ is passed to the backbone, and the following expression is obtained:

(4) $$V_{0}^{\prime }=V_{0}+\gamma E_{C}\left[r\right],$$

where $V_{0}$ is represented as an initial backbone feature and γ is a balancing module hyperparameter, and when γ=0.6, the effect is the best. Through the incorporation of modules, camera clustering is completed to minimize the impact of camera differences.

(2) Cross-level Feature Extraction Module

**Figure 6:**
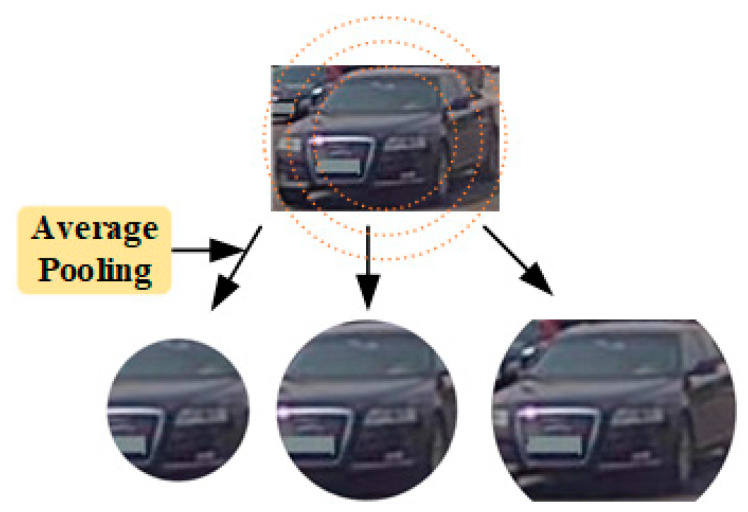
Flowchart of the cross-level feature extraction module.

The vehicle’s center point coordinates (x, y) are obtained through detection, and then the object Re-ID feature centered at (x, y) is extracted from the feature map to obtain the vehicle’s frame feature. After observing the majority of vehicle frames, the most distinctive features (logo, headlights, etc.) are centered. As shown in Figure 6, as the receptive field expands, the vehicle’s distinguishing characteristics increase, but so does the amount of background information, which contains more difficult-to-distinguish information. A novel form of progressive central pooling is introduced to process extracted features hierarchically.

To implement the preceding statement, local characteristics must first be hierarchically set. Figure 6 is focused on the initial pooling center region, which is followed by decreasing levels. In the context of hierarchical modules, the information contained in the vehicle’s features is increased from less to more, from concentrated to generalized, resulting in more generalized training. Assuming that the lower left corner is the origin of the image $I\in R^{W\times H}$, the circular center mask region M of the k region can be expressed as follows:

(5) $$M_{x,y}^{k}=\left\{\begin{array}{cc} 10 & if{\left(x-\frac{W}{2}\right)}^{2}+{\left(y-\frac{H}{2}\right)}^{2}\leq {R_{k}}^{2}\\ 0 & otherwise \end{array}\right.$$

where $R_{k}$ is represented as the radius on the kth circle. The extracted mask features are then utilized to reproject the features. The final Re-ID features are acquired.

(3) Re-ID Loss Calculation

The network is optimized by building global feature OIM loss [12] (Online Instance Matching loss) and Triplet loss [22]. OIM loss is a kind of loss proposed for pedestrian search tasks. Its role is to store all the feature centers that mark identities in a lookup table (LUT). $V\in R^{D\times L}=\{v_{1},\ldots ,v_{L}\}$ represents L D-dimensional feature vectors. In addition, a circular list is compiled of Q unlabeled identity features, $U\in R^{D\times Q}=\{u_{1},\ldots ,u_{Q}\}$. The following formula is used to calculate the probability of identifying x as the identity with ID i based on the two vectors presented above:

(6) $$p_{i}=\frac{\exp\left(v_{i}^{T}x/\tau \right)}{\sum_{j=1}^{L}\exp\left(v_{j}^{T}x/\tau \right)+\sum_{k=1}^{Q}\exp\left(u_{k}^{T}x/\tau \right)}$$

where *T* is represented as transpose. The objective of OIM is to minimize the expected probability of a negative logarithm:

(7) $$L_{OIM}=-E_{x}\left[\log p_{t}\right]$$

Then, the commonly used triple loss function is added in Re-ID [22] to distinguish the detailed features between classes, shorten the distance with the corresponding features stored in the LUT, and push the distance of the features outside the LUT to a great distance. After detection, first the candidate feature set is obtained, and then the ternary combination set {a,p,n} is set. Consequently, the triplet loss function $L_{tri}$ is as follows:

(8) $$L_{tri}=\log\left[1+\exp\left({\left\|f_{a}-f_{p}\right\|}_{2}^{2}-{\left\|f_{a}-f_{n}\right\|}_{2}^{2}\right)\right]$$

where $f_{a}$ is represented as the anchor feature itself, $f_{p}$ is represented as the positive sample feature with the same ID as an anchor, and $f_{n}$ is the feature with a different ID than anchor. Finally, the Re-ID branch’s computational loss is as follows:

(9) $$L_{Reid}=L_{OIM}+\lambda L_{tri}$$

when λ=0.6, the effect is the best.

## 5. Experiments and Results

### 5.1. Experiment Setting

Datasets: Extensive experiments were conducted on the DAIR-V2XSearch dataset. Since there is no existing vehicle search dataset, the popular pedestrian search dataset PRW [1] was chosen to test the effectiveness and generalizability of the proposed method. The PRW dataset includes images captured by six roadside cameras on a college campus. The data is sampled from videos, and pedestrian identities and bounding boxes are manually labeled. This dataset is used to validate the model’s generalizability. The data annotations for the two datasets are displayed in Table 1.

Backbone: ResNet-50 [33] is the backbone for feature extraction. The weights trained by ImageNet [34] are utilized as the pre-trained model, and the number of layers is reduced after the pooling layer and an ibn-a block are added [35].

Implementation Details: Among other techniques, resize, random erase, horizontal flip, and mixup are used to enhance the data. For network training, 80 epochs are assigned. The SGD optimizer is employed to expedite the model’s approach to the optimal solution; its momentum is set to 0.9, and its weight decays to 1 × 10^−4^. Using cosine annealing, the learning rate of the optimizer is set in the range of 7.7 × 10^−5^ to 1 × 10^−2^ for the first 20 epochs, remains at 1 × 10^−2^ for the next 20 to 60 epochs, and then decreases to 7.7 × 10^−5^ for the remaining epochs of the training process.

Evaluation index: Mean average precision (mAP) [36] and cumulative matching characteristics (CMC) [37] are used for testing to determine the effectiveness of the proposed network in solving the vehicle search problem after the training phase. mAP is used to evaluate Re-ID’s overall performance. CMC is represented as the precision of query flags that appear on candidate lists of various sizes. Recall and AP are utilized to evaluate a detector’s performance. In addition, PRW is employed to validate the generalization performance of the model.

Training: The deep learning framework Pytorch 1.8 and the GPU NVIDIA RTX 2080 Ti are employed for all of our training experiments. The batch size for training is set to 4. Using the same GPU training dataset, DAIR-V2XSearch requires four hours to be trained, while PRW requires six hours.

### 5.2. Ablation Experiments

(1) Performance Analysis of Each Module

As shown in Table 2, ablation experiments are conducted on the DAIR-V2XSearch and PRW datasets to determine the efficacy of each module.

Baseline: As the baseline network, the YOLOX model is added with a Re-ID head in parallel with the detection head. As shown in Table 2, the baseline is offered enhancements by the various modules we have created. In DAIR-V2XSearch and PRW, all modules are combined and compared to the baseline; Rank-1 is improved by 4.95% and 3.16%, while mAP is improved by 6.22% and 5.4747%, respectively.

Comparison of different FPN levels: To evaluate the impact of FPN scale alignment, different levels of feature maps are created, and results are presented in Table 3. Particularly, the characteristics of $P_{3}$, $P_{4}$, and $P_{5}$ are evaluated with 8, 16, and 32 strides, respectively. Comparing the detection accuracy to the Re-ID accuracy, the maximum receptive field feature $P_{3}$ would result in the highest accuracy.

Comparison under varying numbers of FPN branches: To evaluate the impact of varying numbers of FPN branches on the Re-ID task, a number of comparisons are designed. The $\left\{P_{3},P_{4}\right\}$ *P* size range is particular set to [0, 128] and [128, ∞], and the $\left\{P_{3},P_{4},P_{5}\right\}$ *P* size range is particular set to [0, 128], [128, 256], and [256, ∞]. As shown in Table 4, the increase in the number of FPN branches improves the detection recall rate, but reduces the Re-ID accuracy to some degree.

Influence of CGM at Various Stages: In Table 5, the influence of CGM is examined at varying stages of ResNet-50 precision. The PRW and DAIR-V2XSearch datasets are validated by us at stage 2 for optimal performance.

**Figure 7:**
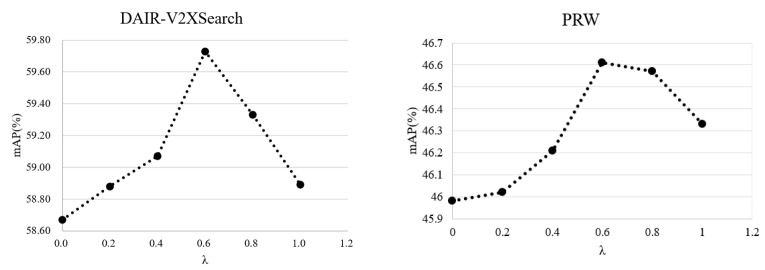
Comparison charts of two datasets under different λ precision line.

The effect of various coefficients of the ternary ID’s loss function: The impact of various coefficients on the precision of Re-ID is investigated. As shown in Figure 7, for the two datasets, the effect of the model is improved differently depending on the coefficients, but overall, there is no significant difference in its effectiveness. The optimal results are achieved when λ = 0.6.

(2) Visualized Analysis

**Figure 8:**
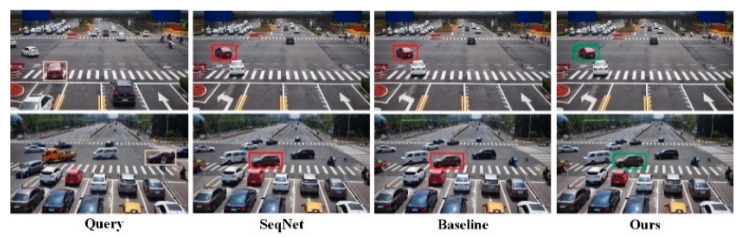
Rank-1 search results from the gallery in the DAIR-V2XSearch dataset corresponding to the query image. The yellow box represents the original data. The red box represents the output result error. The green box represents the output result error.

Visualization of retrieval results. Figure 8 demonstrates the efficacy of the proposed network by displaying the Rank-1 results of baseline and PFE-Net. Orange boxes are represented as the target of the query, as opposed to green for correct results and red for incorrect results. The results demonstrate that our method is more precise.

**Figure 9:**
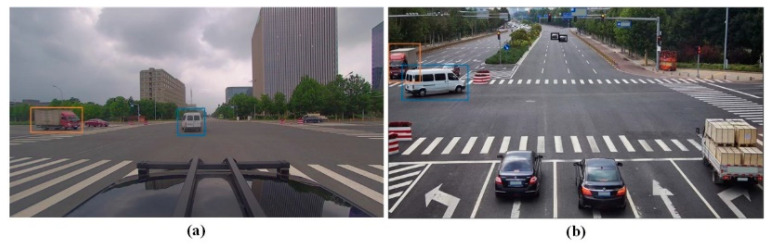
Visualization of the simultaneous perceptual effect of two cameras. The box color of the same vehicle is the same. (**a**) Shoot for the vehicle camera. (**b**) Shoot for the roadside camera.

Visualizing perceptual effects across cameras. As depicted in Figure 9, the correct model results are inserted into the original image for the purpose of effect comparison, which is the simultaneous shooting situation of both devices. The results of data collection from the perspective of a single vehicle are shown in Figure 9a. Only two vehicles can be seen from this vantage point, and the road conditions ahead cannot be determined. However, with the addition of Figure 9b, the receptive field of road conditions expands, and road conditions for more than two vehicles can be obtained. By matching the two devices, the perception limitations of a single vehicle are eliminated, enabling the completion of tasks such as road condition evaluation and route planning.

### 5.3. Comparisons with the State of the Art

Our model is compared to current mainstream methods (including the one-step model [9,10] and the two-step model [38]) on two vehicle search benchmarks, PRW and DAIR-V2XSearch, and finds that it performs well.

Analysis of the results of DAIR-V2XSearch: The new dataset is validated using the previously proposed partial method. As shown in Table 6, the staged feature extraction network is outperformed by all one-step detection models. In contrast, the one-stage, anchor-free detection framework is employed, which can be calculated more quickly.

PRW result analysis: PRW is a pedestrian search dataset. The proposed network is incorporated into this dataset in order to validate generalizations. As demonstrated in line 2 of Table 6, our model is comparable to the vast majority of existing algorithms.

Efficiency Comparison: The efficiency of our model is compared to that of existing networks. As before, our code is implemented using PyTorch, and the input image size is adjusted to 900 × 1500 pixels to ensure consistency. As shown in Table 7, our method has the fastest computation speed, whereas the COAT method is slow and memory-intensive, rendering it unsuitable for tasks such as subsequent deployment despite its high accuracy.

## 6. Conclusions

A vehicle search problem is investigated in this paper in an effort to enable over-the-horizon sensing in autonomous driving. To address the lack of a vehicle search dataset in existing research, the occlusion problem, and to achieve comprehensive perception, DAIR-V2XSearch, a new cross-camera vehicle search dataset in real-world car scenes, is presented. At the same time, a new network is proposed for vehicle search: phased feature extraction networks (PEF-Net), which are used to solve the cross-camera vehicle search task. Considering the problems inherent in the vehicle itself, a cross-level feature aggregation module is also designed, which makes the model more sensitive to the subtle vehicle features and improves the training accuracy of the model. Numerous experiments demonstrate the generalizability of the method. In the future, research will continue to be conducted to improve the accuracy of the method, and at the same time, the research will be put into action to determine the practicability of the method. We believe that this technology can be applied to subsequent perception tasks like object tracking and trajectory prediction, and it will be increasingly advantageous for autonomous driving tasks like control and decision-making.

## Figures and Tables

**Table 1:** Data comparison between two datasets.

	DAIR-V2XSearch	PRW
Frame	4367	11,816
ID	492	932
Annotated	23,871	34,304
Box per ID	48.5	36.8
Gallery box	7826	100–500 k
Camera	2	6

**Table 2:** Comparison of the precision of PRW and DAIR-V2XSearch datasets for distinct modules. √ represents that the module is used. × represents that the module is not used.

Methods			PRW		DAIR-V2XSearch	
FPN	CGM	CFE	mAP	Rank-1	mAP	Rank-1
×	×	×	43.45	81.53	54.78	89.33
√	×	×	43.61	80.80	55.54	92.55
×	√	×	44.20	81.88	54.80	93.64
×	×	√	43.80	82.70	55.20	92.55
√	√	×	45.25	83.48	56.30	94.40
√	×	√	44.60	81.70	55.30	93.35
×	√	√	45.10	84.77	56.54	94.00
√	√	√	46.61	86.60	59.73	95.95

**Table 3:** Comparison of the FPN levels in the DAIR-V2XSearch dataset.

Methods	Detection		Re-ID	
	Recall	AP	mAP	Rank-1
$P_{3}$	97.16	87.56	59.73	95.95
$P_{4}$	95.73	89.60	59.10	95.55
$P_{5}$	93.86	84.33	58.50	94.84

**Table 4:** Effect of the number of FPN branches on the precision of the DAIR-V2XSearch dataset.

Methods	Detection		Re-ID	
	Recall	AP	mAP	Rank-1
$P_{3}$	97.16	87.56	59.73	95.95
$P_{3},P_{4}$	97.12	84.84	58.98	94.55
$P_{3},P_{4},P_{5}$	97.31	86.48	57.50	93.73

**Table 5:** Comparison of the Re-ID precision of CGM at various stages.

Stage	PRW		DAIR-V2XSearch	
	mAP	Rank-1	mAP	Rank-1
No	44.60	81.70	55.30	93.35
Stage 1	45.87	84.61	58.15	94.73
Stage 2	46.61	86.60	59.73	95.95
Stage 3	46.27	83.89	57.94	95.51
Stage 4	44.61	82.42	56.16	93.94

**Table 6:** Comparison of PRW and DAIR-V2XSearch with the most advanced methods.

Methods		DAIR-V2XSearch		PRW	
		mAP	Rank-1	mAP	Rank-1
Two-Step	DPM [1]	-	-	20.5	48.3
	MGTS [39]	-	-	32.6	72.1
	RDLR [40]	-	-	42.9	70.2
	IGPN [9]	-	-	47.2	87.0
	TCTS [10]	-	-	46.8	87.5
One-Step	OIM [12]	-	-	21.3	49.9
	IAN [41]	-	-	23.0	61.9
	HOIM [42]	-	-	39.8	80.4
	APNet [43]	-	-	41.9	81.4
	NAE [11]	-	-	43.3	80.9
	NAE+ [11]	-	-	44.0	81.1
	AlignPS [18]	53.27	88.48	45.9	81.9
	SeqNet [38]	54.45	89.55	46.7	83.4
	AGWF [44]	-	-	53.3	87.7
	COAT [13]			54.0	89.1
	Baseline	54.78	89.33	43.45	81.53
	Ours	59.73	95.95	46.61	86.60

**Table 7:** FPS comparison of each model.

Methods	FPS
NAE	14.48
AlignPS	16.39
COAT	11.14
Ours	16.40

## Data Availability

Not applicable.
